# Antegrade anterior column acetabulum fracture fixation with cannulated compression headless screws—A biomechanical study on standardized osteoporotic artificial bone

**DOI:** 10.1371/journal.pone.0300256

**Published:** 2024-06-03

**Authors:** Till Berk, Ivan Zderic, Peter Schwarzenberg, Torsten Pastor, Ludmil Drenchev, Hristo Kostov Skulev, Geoff Richards, Christian Hierholzer, Sascha Halvachizadeh, Hans-Christoph Pape, Boyko Gueorguiev

**Affiliations:** 1 AO Research Institute Davos, Davos, Switzerland; 2 Department of Trauma, University Hospital Zurich, Zurich, Switzerland; 3 Department of Orthopedic and Trauma Surgery, Lucerne Cantonal Hospital, Lucerne, Switzerland; 4 Bulgarian Academy of Sciences, Institute of Metal Science ’’Acad. A. Balevski’’, Sofia, Bulgaria; 5 Harald-Tscherne Laboratory for Orthopedic and Trauma Research, University of Zurich, Zurich, Switzerland; Assiut University Faculty of Medicine, EGYPT

## Abstract

**Purpose:**

Due to the increase in life expectancy and high-energy traumas, anterior column acetabular fractures (ACFs) are also increasing. While open reduction and internal fixation (ORIF) is still the standard surgical procedure, minimally invasive, percutaneous fixation of osteoporotic acetabulum fractures (AF) are growing in popularity. The aim of this biomechanical study was to evaluate the biomechanical competence following antegrade fixation with a standard screw versus a cannulated compression headless screw.

**Methods:**

Eight anatomical osteoporotic composite pelvises were given an anterior column fracture. Two groups of eight specimens each (n = 8) for fixation with either a 6.5 mm cannulated compression headless screw in group Anterior Acetabulum Canulated Compression Headless Screw (AACCH), or with a 6.5 mm partially threaded cannulated screw in group Anterior Acetabulum Standard Screw (AASS) where compared. Each specimen was biomechanically loaded cyclically at a rate of 2 Hz with monotonically increasing compressive load until failure. Motions were assessed by means of optical motion tracking.

**Results:**

Initial construct stiffness trended higher in group AACCH at 152.4 ± 23.1 N/mm compared to group AASS at 118.5 ± 34.3 N/mm, p = 0.051. Numbers of cycles and corresponding peak load at failure, were significantly higher in group AACCH at 6734 ± 1669 cycles and 873.4 ± 166.9 N versus group AASS at 4440 ± 2063 cycles and 644.0 ± 206.3 N, p = 0.041. Failure modes were breakout of the screws around the proximal entry point.

**Conclusion:**

From a biomechanical perspective, group AACCH was associated with superior biomechanical competence compared to standard partially threaded cannulated screws and could therefore be considered as valid alternative for fixation of anterior acetabulum fractures.

## Introduction

Due to the increase in life expectancies of geriatric patients and high-energy traumas, anterior column acetabular fractures (ACFs) are also increasing [1–3]. Minimally invasive percutaneous fixation of acetabulum fractures (AF) are still increasing in popularity [3–5], however, open reduction and internal fixation (ORIF) is still the standard surgical procedure for the treatment of displaced AF or fracture morphology concerning the weight-bearing dome [3, 6, 7]. Known complications due to the surgical approach include wound infections, blood loss, heterotopic ossifications and iatrogenic injuries to blood vessels or nerves and occur in up to 25% of cases [6, 8–10]. However, percutaneous screw fixation (PSF) has been promoted for treating non-displaced or minimally displaced ACF [11, 12]. In elderly patients (> 60 years), AFs with a displaced anterior column, account for up to 60,6% of cases [1]. Percutaneous fixation has the potential to be a safe and effective option in old and frail patients [13]. PSFs of the anterior column of the acetabulum are routinely approached in either an antegrade or retrograde fashion and have been proven to maintain satisfactory stability [11, 14–16]. With a transition towards minimally invasive therapies, implant design adaptation remains the subject of recent research and could further improve the postoperative outcome.

Purpose: With regard to minimally invasive, percutaneous screw fixation of ACF in osteoporotic specimens, the aim of this biomechanical study was to evaluate the biomechanical competence following antegrade fixation with standard versus cannulated compression headless screws (AACCH). To our knowledge, the usage of AACCH for ACF has never been investigated.

## Materials and methods

Eight anatomical osteoporotic composite pelvises were used in this study (Model LSS4055®, Synbone, Zizers, Switzerland). The chosen fractures were an anterior column fracture according to the Letournel classification [17]. The acetabulum fracture was generated by an osteotomy with a 1 mm bone sawblade as well as a custom template to ensure identical osteotomies (fractures). Each pelvis was considered for testing on both the left and right side. Therefore, sixteen hemi-pelvis constructs were available and stratified into two groups of eight specimens each (n = 8) corresponding to their treatment as follows:

**Group AACCH**: stabilization of the ACF was achieved using cannulated AACCH screws 6.5 mm: partial threaded, 90 mm in length.

**Group AASS**: stabilization of the ACF was achieved using standard cannulated screws 6.5 mm, partial threaded, 90 mm in length.

The screws in group AASS were made of stainless steel (316L), whereas the screws in group AACCH was made of titanium alloy (Ti-6Al-4V ELI). The same manufacturer (DePuy Synthes, Zuchwil, Switzerland) provided all implants. The two groups had equal samples of left and right AFs.

### Surgical procedure

**Fig 1:**
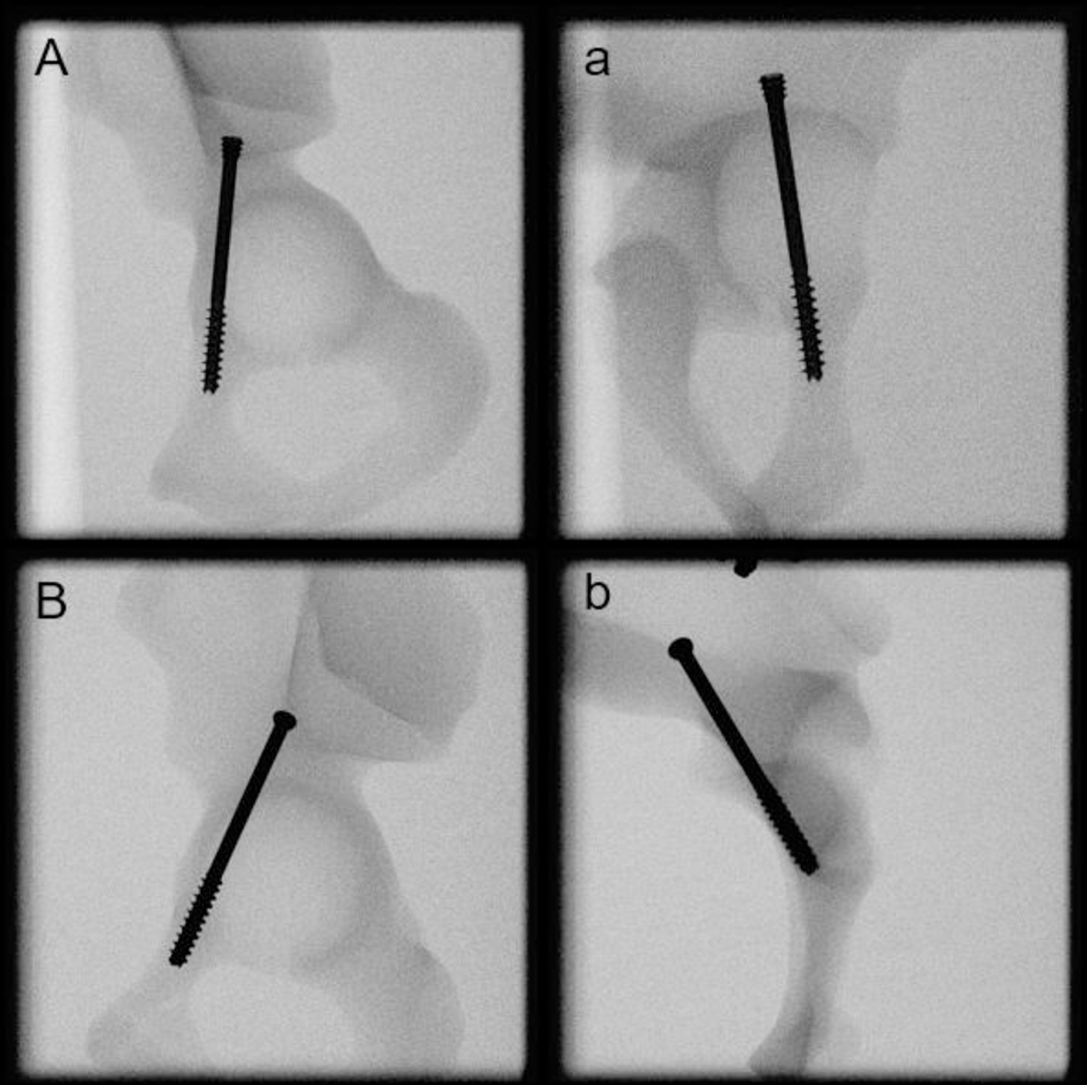
X-rays after instrumentation showing exemplified specimens from group AACCH (a), AASS (b).

Following anatomical reduction of the ACF, a 2.8 mm guide wire was placed in the acetabulum across the fracture and cephalad to the superior pubic ramus according to the AO surgery reference in an antegrade fashion [18]. The starting point for the Kirschner (K) -wire placement was radiologically determined at the proximal Os ilium. The K-wire placement was constantly being checked under fluoroscopy, avoiding any perforations, via falsa, or cortical disruptions that could influence the outcome. Following pilot drilling, the cannulated screws were inserted over the K-wires and tightened according to the operator’s best practice. The head of the conventional screws was not countersunk as in the CCH screws, but placed flush on the proximal cortex. All procedures were implemented following the surgical guidelines of the implant manufacturer as well as following the AO surgery reference and recommendations [18]. An experienced surgeon with senior attending status performed all procedures. After instrumentation, AP, obturator oblique, inlet views, and iliac oblique views were attained via X-rays for final documentation and verification (Fig 1).

### Biomechanical testing

**Fig 2:**
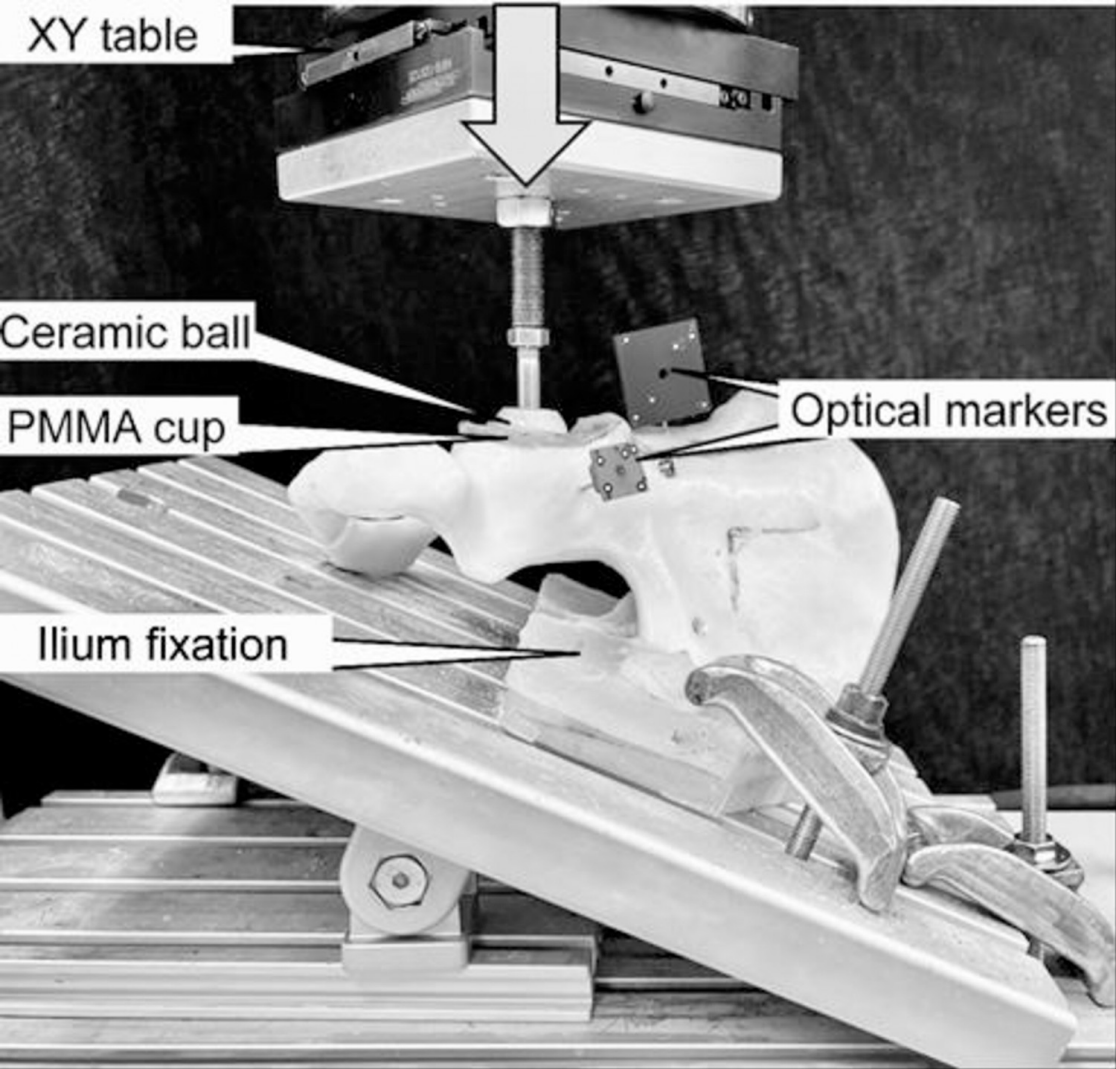
Test setup with a specimen mounted for biomechanical testing. Vertical arrow denotes loading direction.

Biomechanical testing was performed on a servohydraulic material test system (Mini Bionix II 858; MTS Systems, Eden Prairie, MN, USA) equipped with a 4 kN load cell (HUPPERT 6, HUPPERT GmbH, Herrenberg, Germany). The setup was adopted from previous biomechanical studies investigating acetabulum fractures [19] (Fig 2). Each hemi-pelvic was tested and aligned in an inverted upright standing position. To achieve this, the specimen rested on an aluminum base plate, which was firmly connected to the machine base, and inclined by 20° in the coronal plane, following the protocol of Morosato et al. [20] for positioning the medial aspect of the symphysis as well as the sacroiliac joint flush with the base plate. The sacroiliac joint was further constrained to the base plate via two molded polymethylmethacrylate (PMMA, SCS-Beracryl D-28, Suter Kunststoffe AG/Swiss-Composite, Fraubrunnen, Switzerland) blocks, which allowed a consistent mounting of all specimens.

Axial compression along the machine axis was applied to the acetabulum via a ceramic ball (28 mm radius). A homogenous load transfer to the specimens was achieved by a molded PMMA hemispherical cavity resting in the acetabulum. This configuration simulated a hip joint reaction force trajectory during walking, as previously described by Bergmann et al. [21].

The loading protocol commenced with an initial nondestructive quasi-static ramp from 20 N preload to 200 N at a rate of 18 N/s, followed by progressively increasing cyclic loading in axial compression with a physiological profile of each cycle at a rate of 2 Hz [21]. While keeping the valley load at a constant level of 20 N, the peak load, starting at 200 N, was monotonically increased cycle by cycle at a rate of 0.1 N/cycle until the test stop criterion of 10 mm actuator displacement had been achieved with respect to its position at the beginning of the loading protocol, which was found adequate to provoke catastrophic failure of the specimens [22, 23].

### Data acquisition & analysis

Machine data of axial displacement and axial load were continuously acquired from the machine transducer and load cell throughout the tests at 200 Hz. Based on these, the construct stiffness was calculated from the ascending load-displacement curve of the initial quasi-static ramp within the linear loading range between 80 N and 180 N.

The relative displacements between the fractured fragments were continuously assessed at 20 Hz throughout the tests in all six degrees of freedom by means of optical motion tracking. Therefore, individual marker sets, consisting of multiple single optical markers, were attached to the superior and inferior fragments adjacent to the fracture line using K-wires. A local anatomical coordinate system was generated based on proper alignment of the superior marker set. A second coordinate system was created based on the fracture plane, which was virtually defined using a dedicated touch probe. The coordinates of the markers were then tracked with a stereographic optical camera system (Aramis SRX, Carl Zeiss GOM Metrology GmbH, Braunschweig, Germany) within these two coordinate systems. Based on these measurements, the relative movement of the two bone fragments were calculated as the Euclidean normal distance of the translational displacements along the three principal axes and defined as total posterior displacement and total inferior displacement. Additionally, the combined angular displacement was calculated as the gap opening between the two initially reduced osteotomy/fracture surfaces adjoining each other in the fracture gap and defined as gap angle. Furthermore, the angular displacement between the fragments in the fracture plane was assessed and defined as torsional displacement.

The outcome measures were calculated at five intermediate time points of cyclic testing after 1000, 2000, 3000, 4000, and 5000 test cycles. The latter represented the highest rounded number of cycles at which none of the specimens had failed and dropouts could not artifactually influence the results. The values were considered with respect to the values at the beginning of the cyclic test and were calculated in peak loading condition. Also, the criterion for specimen failure was set at 3 mm total displacement, and the corresponding number of cycles until fulfillment of this criterion were calculated.

Statistical analysis among the outcome measures was performed with SPSS software (v.27, IBM SPSS, Armonk, NY, USA). General Linear Model Repeated Measures test with Bonferroni post-hoc test for multiple comparisons were conducted to determine significant differences between the treatment groups for the outcome measures of the parameters of interest evaluated over the time points during cyclic testing after 1000, 2000, 3000, 4000, and 5000 cycles. Mean and standard deviations were calculated for each parameter of interest and group separately. Initial axial construct stiffness and the numbers of cycles for the different failure criteria, including cycles to earliest failure, were compared among the groups with One-Way Analysis of Variance (ANOVA) considering either Bonferroni or Games-Howell post-poc tests. Level of significance was set at 0.05 for all statistical tests.

## Results

Initial construct stiffness was 152.4 ± 23.1 N/mm for group AACCH and 118.5 ± 34.3 N/mm for group AASS, which trended towards higher stiffness values in group AACCH, p = 0.051.

**Fig 3:**
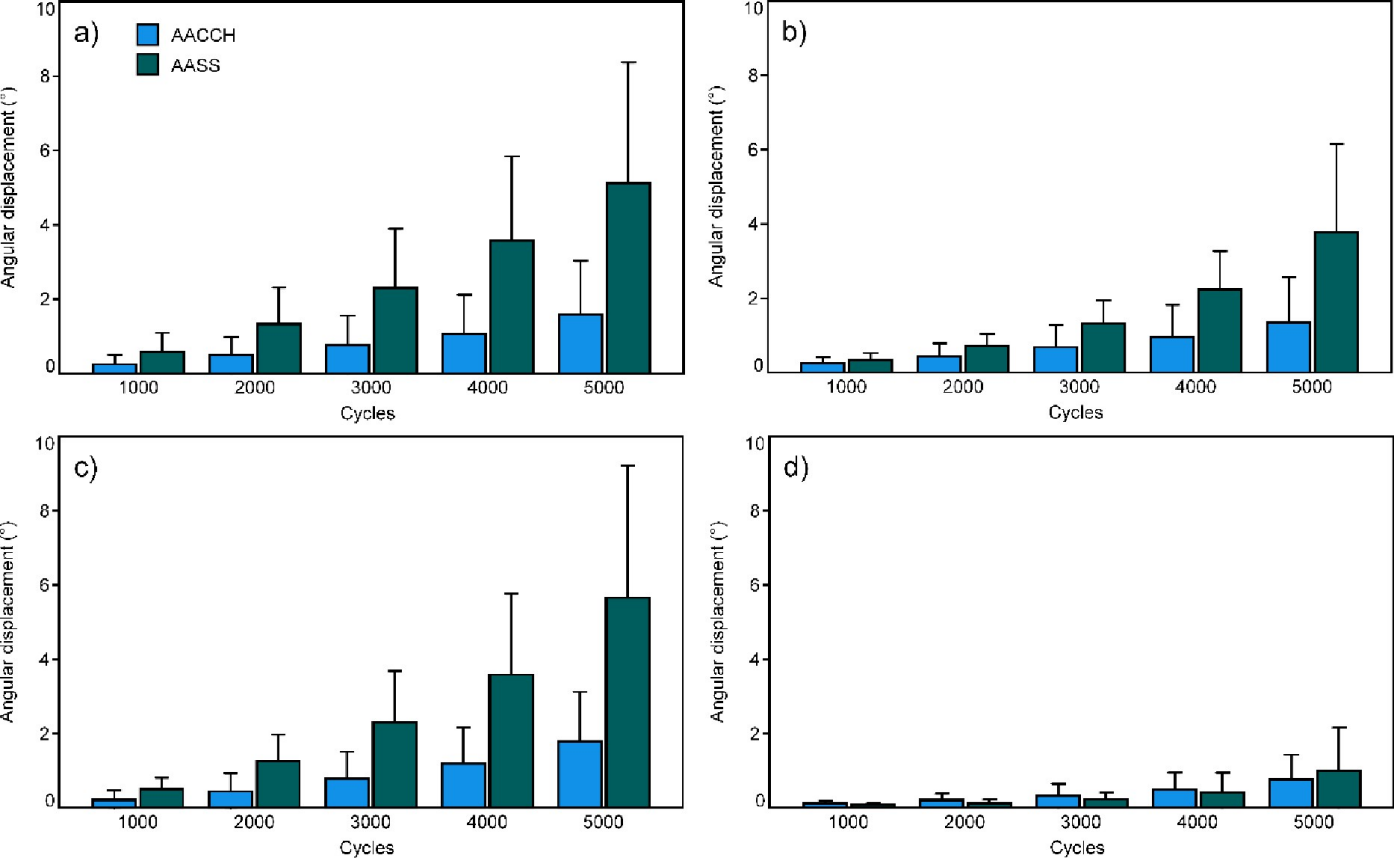
Total displacement posterior (a) and inferior (b), gap angle (c), and torsion (d), over the investigated five time points during cyclic loading after 1000, 2000, 3000, 4000, and 5000, test cycles, shown for each group separately in terms of mean and SD.

Outcome measures of the parameters of interest investigated over the five time points, total displacement anterior, total displacement posterior, gap angle, and torsion, are summarized in Fig 3. The outcomes of each parameter were associated with significantly higher values for AACCH versus AASS for total displacement posterior (p = 0.031), total displacement inferior (p = 0.023), and gap angle (p = 0.016), but not for torsion (p = 0.945).

**Fig 4:**
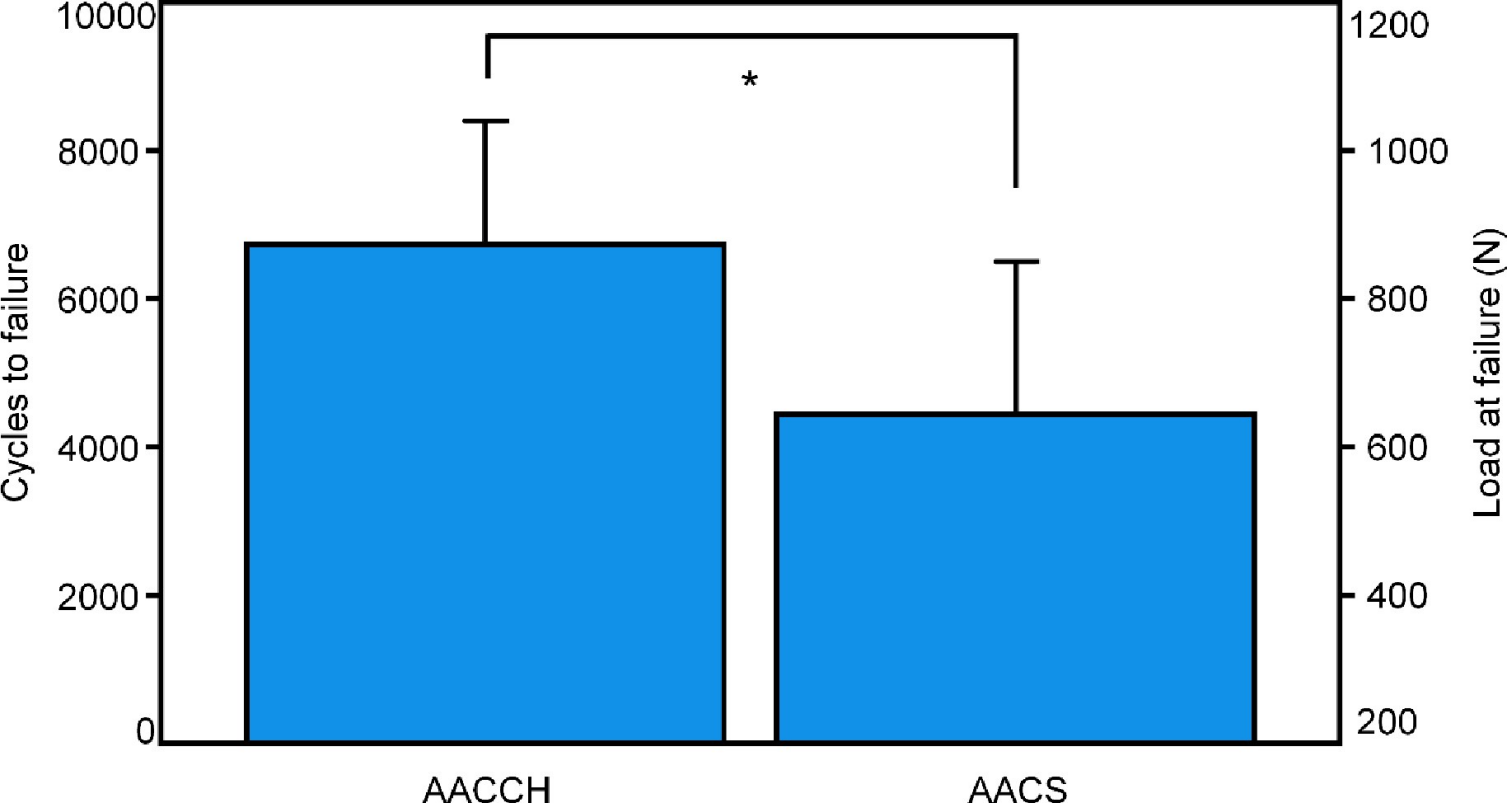
Cycles to failure and corresponding load at failure shown for each group separately in terms of mean and SD. Star indicates significant difference.

The numbers of cycles, and the corresponding peak load at failure (3 mm total displacement), were 6734 ± 1669 and 873.4 ± 166.9 N respectively for group AACCH, and 4440 ± 2063 and 644.0 ± 206.3 N for group AASS, with significantly higher values for AACCH, p = 0.041 (Fig 4).

The failure modes were expressed by a breakout of the screws around the proximal entry point or by a fracture dislocation with a dislocation of the PMMA inlay from the acetabulum. There was an comparable distribution of failures in both groups.

## Discussion

The aim of this biomechanical study was to evaluate the primary stability of minimally invasive, percutaneous, antegrade screw fixation of anterior column acetabulum fractures. For this purpose, a standard cannulated screw was compared to an AACCH. The following three main points can be concluded:

1. Initial construct stiffness trended towards a higher value in group AACCH when compared to group AASS.
2. Significantly higher values for group AACCH versus group AASS were found for total anterior and posterior displacement and gap angle.
3. The numbers of cycles and the corresponding peak load at failure for 3 mm total displacement showed significantly higher values for group AACCH versus group AASS.

A comparable study investigated the strength of different screw types in artificial bone specimens of ACF in comparison to standard plate fixation [24]. They concluded that titanium screws were equivalent to plate fixation and that stainless steel and Poly-l-Lactid screw fixations were equal to each other but inferior to titanium [24]. These findings are comparable to the findings in this investigation; the titanium AACCH showed overall superior results when compared to stainless steel screws in group AASS. However, no screw breakages appeared in any of the tests. Catastrophic failure occurred in form of breakouts of the screws around the proximal entry point or by fracture dislocation of the acetabulum. These differences might base on both, the screw design and the screw material. Unfortunately, screws of the same material were not available to correct for the potential influence of material on the biomechanical testing. From a clinical point of view, the impact of routinely used screw material on mechanical stability is minimal, as both materials are utilized equally without evidence of superiority of one material above another [12, 25, 26].

Therefore, view of the results, the design seems to be of greater significance than the material. Nonetheless, the question of the stronger material cannot be answered conclusively in the current study.

Regarding the chosen size of the screws used in this investigation, there is potential for discussion. 6.5mm or 7.3mm cannulated screw are standard diameter for screw fixation of acetabular column fractures. 7.3 mm screws were used in a previous study for the retrograde fixation of anterior column fractures and no screw failures were reported [12]. Furthermore, two comparable studies stated that a 8.0 mm screw would also be suitable [15, 25]. Yet, AO surgery references recommend a screw with a diameter over 6.5 mm for PSF in ACF [18]. In the clinical setting, the surgeon might be forced to use smaller diameter screws based on the anatomic properties of the patients`pelvis. The goal of maximal stability might be reached with increased screw diameter.

A clinical study investigated closed reduction and percutaneous screw fixation with 6.5-mm or 8.0-mm cannulated screw for the treatment of anterior column or anterior column-posterior hemi-transverse acetabulum fractures. No loss of reduction in either screw groups was diagnosed [15]. Nevertheless, the investigated cohort of 28 patients must be considered small.

In contrast, a different study concluded by investigating CT scan data for retrograde ACF screw placement-planning in Asian pelvises, that in male patients a 6.5 mm lag screw would fit very well, but the same screw size in females might perforate the bony margin and its use should be carefully evaluated [16].

Concerning senior patients, percutaneous fixation might be a treatment option.It was reported that a loss of reduction is related with the development of posttraumatic arthritis, and hence with poor patient outcome [27]. A more precise reduction might be achieved in the future with the help of new technologies such as patient specific instrumentation, which could have already been shown in fracture fixation [28].

It was shown that open reduction and internal fixation can lead to interfering scar tissue and hardware, which can significantly complicate a hip joint replacement surgery [29].

There are little reports assessing the effect of the type of threading used in ACF screw fixation. Most authors describe partially threaded screw in their investigation/recommendation [12, 18, 25, 26]. It has further been stated, that the choice of the threading should depend on bone quality, patients age and constitution, and fracture morphology [30, 31]. For this reason, only partially threaded screws were used for this investigation.

In the standard screw fixation, the head of the conventional screw was not countersunk in the bone, but rather it was placed flush on the proximal cortical bone. Therefore, the head of the standard screw protrudes from the bone, which could lead to irritation of musculature or tendon structures. With the countersunk CCHS screw, irritation at the surface of the screw is much less likely. However, overgrowth of new bone over the screw head could make possible material removal much more difficult compared to conventional screw.

The titanium screws in group AACCH were more than twice as expensive at the affiliated hospital as the standard steel screws in group AASS. This may considerably complicate the clinical applicability of the screws despite the impressive results presented. One could argue that due to a higher stability, re-operation rates should be significantly lower, and the outcome could be more favorable for patients as a result. However, in times of case-based reimbursements, this seems to little effect on hospital financial-management. Lastly, another aspect to take into consideration is that the proximal end of the CCH screws is embedded in the bone. This end can ossify as the bone heals. This could potentially make the removal of the material more difficult and significantly increase the duration of the operation and the radiation exposure.

Following the present data, no difference in displacement after torsional forces were seen. This might base on the unique anatomic properties of the acetabulum that appears to be more robust against torsional forces. Both screws are comparable regarding torsional forces.

### Strength & limitations

The prominent limitation of this study is the chosen artificial bone specimen that has well known limitations in comparison to cadaver bones. Nevertheless, since we conducted an investigational study with a first step peerless approach, for which there is very little data available, the authors agreed on this approach. In the opinion of the authors, it is ethically justifiable to proceed with a cadaver study in a follow-up analysis. On the other hand, synthetic bone specimens are commonly as well as efficiently used in various biomechanical studies, especially concerning the pelvis [32–36]. Additionally, the poor availability of cadavers can limit the sample size for biomechanical experimentations, and, it is known, that sample sizes used in previous cadaveric publications are in general small [37]. Yet, artificial pelvises allow a standardized and well comparable sample group, which can overpower the many variations in bone quality, which can be observed in human cadaver specimens and are even more cost-effective [36, 38–40]. To this end, the usage of artificial bone models minimizes the variability of test results between test samples [34, 41]. Lastly, the screws compared were manufactured from different materials, which one could argue, could also influence the results. To the knowledge of the authors, the steel screws used are standard implants in trauma care and are not available in titanium, which makes a different design of the study impossible. In addition, no breakages of the inserted screws occurred in either of the groups. Therefore, the authors assume that the material could primarily be negligible at the current stage of the investigation. Another limitation of this study could be the countersinking of the head of the CCHS screws, which leads to a deeper screw position in the bone. In contrast, the head of the standard screw is positioned flush with the proximal cortical bone, which allows the screw to penetrate less deeply into the bone. Whether the specific design of the CCHS screw with its additional anchoring in the bone or the increased length of the screw in the region of the superior pubic branch are responsible for the differences obtained in this study cannot be answered in the chosen study design. This would require a repetition of this study with different screw lengths in a direct comparison of the selected screws. However, the question of whether the material itself or the screw design, are responsible for the results found, based on the current data, cannot be answered and should be investigated in further studies.

## Conclusion

From a biomechanical perspective, cannulated compression headless screws were associated with superior biomechanical competence compared to standard partially threaded cannulated screws and could therefore be considered as possible alternative for fixation of anterior acetabulum fractures. Further biomechanical studies should be initiated using cadaver bones and larger sample sizes for a better understanding of AACCH for ACF fixation.
